# Fish, food security and health in Pacific Island countries and territories: a systematic literature review

**DOI:** 10.1186/s12889-016-2953-9

**Published:** 2016-03-24

**Authors:** Karen E. Charlton, Joanna Russell, Emma Gorman, Quentin Hanich, Aurélie Delisle, Brooke Campbell, Johann Bell

**Affiliations:** School of Medicine, University of Wollongong, Wollongong, NSW 2522 Australia; School of Health and Society, University of Wollongong, Wollongong, NSW 2522 Australia; Australian National Centre for Ocean Resources and Security (ANCORS), University of Wollongong, Wollongong, NSW 2522 Australia; Betty and Gordon Moore Center for Science and Oceans, Conservation International, Arlington, VA 22202 USA

**Keywords:** Pacific Islands, Food security, Fish, Systematic review, Non communicable diseases

## Abstract

**Background:**

Pacific Island countries and territories (PICTs) face a double burden of disease, with a high prevalence of household food insecurity and childhood micronutrient deficiencies, accompanied by a burgeoning increase in adult obesity, diabetes and heart disease.

**Methods:**

A systematic literature review was undertaken to assess whether increased availability of, and access to, fish improves a) household food security and b) individual nutritional status.

**Results:**

A total of 29 studies were reviewed. Fourteen studies identified fish as the primary food source for Pacific Islanders and five studies reported fish/seafood as the primary source of dietary protein. Fish consumption varied by cultural sub-region and Pacific Island countries and territories. Fish consumption and nutritional status was addressed in nine studies, reporting moderate iodine deficiency in Vanuatu where only 30 % of participants consumed mostly fresh fish. Similarly, the degree to which Pacific Islanders depended on fishing for household income and livelihood varied between and within PICTs. For more economically developed countries, household income was derived increasingly from salaried work and dependency on fishing activities has been declining.

**Conclusions:**

Fishing remains a major contributor to food security in PICTs, through subsistence production and income generation. However, there is a paucity of research aimed at assessing how maintaining and/or improving fish consumption benefits the diets and health of Pacific Islanders as they contend with the ongoing nutrition transition that is characterised by an increasing demand for packaged imported foods, such as canned meats, instant noodles, cereals, rice, and sugar-sweetened beverages, with subsequent decreased consumption of locally-produced plants and animals.

## Background

The Pacific Island region comprises 22 countries and territories which are diverse in geography, population size, culture and economy. Melanesian island countries (Table 1) are typically large and mountainous with fertile soils, whereas the smaller Polynesian and Micronesian islands (Table 1) are either volcanic areas or low lying coral atolls [1]. Population sizes vary from as little as 1,200 in Tokelau, the smallest Pacific Island territory to 7.4 million in Papua New Guinea, the largest Pacific Island country [2]. GDP per capita is low in most Pacific Island countries and territories (PICTs), ranging from US$1,651 in Kiribati to US$36,405 in New Caledonia [3] with five PICTs (Kiribati, Samoa, Solomon Islands, Tuvalu and Vanuatu) currently classified as Least Developed Countries [1, 4].

**Table 1:** Pacific Island countries and territories

Melanesian	Polynesian	Micronesian
Fiji	American Samoa	Federated States of Micronesia
New Caledonia	Cook Islands	Guam
Papua New Guinea	French Polynesia	Kiribati
Solomon Islands	Niue	Marshall Islands
Vanuatu	Pitcairn Island	Nauru
	Samoa	Northern Marianas Islands
	Tonga	Palau
	Tokelau	
	Tuvalu	
	Wallis and Futuna	

The nutrition transition [5] is well underway in PICTs. Dietary patterns have shifted since the 1970–80s from reliance on traditional low fat diets, typically based on complex carbohydrates, fresh fish and meat and leafy greens, to increasingly modern diets, based on refined starch, oils, processed meats and confectionary [6–10]. This relatively rapid transition in the Pacific Island region has resulted in an increasing demand for packaged imported foods, such as canned meats, instant noodles, cereals, rice, and sugar-sweetened beverages, with subsequent decreased consumption of locally-produced plants and animals, leading to high vulnerability to food insecurity [11]. Moreover, such diets have been identified as a major contributor to the double burden of communicable and non communicable diseases (NCDs) in the region [6, 12]. NCDs now account for between 60 and 80 % of all deaths in the region [13, 14] and the prevalence of diabetes and obesity are among the highest in the world [15]. At the same time, the incidence of malnutrition and vitamin and mineral deficiencies continue to be major public health concerns [16, 17]. Iron-deficiency anaemia, which is associated with impaired cognitive and motor development, low birth weight and prematurity [18, 19], affects up to 57 % of the population in some PICTs (mostly children and women) [20]. As has been reported in other low-income countries [21–26], it is not uncommon to find both malnourished children and overweight/obese adults co-residing in the same household [12].

Food security, defined as the physical, social and economic ability to access sufficient, safe and nutritious food, is already identified as under threat in many PICTs due to the dietary shifts caused by changes in population growth, urbanisation and climate [1, 27]. It is evident that significant economic, environmental and population health reforms will be required to ensure that Pacific Island populations have reliable sources of affordable and nutritious food in the future [28].

Across the globe, fish and fisheries have been identified as being a crucial element in achieving food security, particularly in less-developed countries [29, 30]. In the Pacific Island region, the major contribution of fisheries to livelihoods, revenue and development is undisputed. Fish contribute substantially to both the subsistence and market-based economies of PICTs, and national rates of fish consumption are among the highest in the world [27]. It is acknowledged that increased fish supplies are needed to meet growing food security demands [31]. However, there is a lack of information regarding the contribution of fish consumption to overall nutritional adequacy and health status in PICT populations.

Here, we report the findings of a systematic literature review undertaken to explore how availability of fresh fish affects the nutritional status of Pacific Island populations. The review considered both direct benefits of fish consumption to nutritional status, as well as indirect benefits to nutritional status and food security resulting from improvements in livelihoods related to fishing.

## Methods

The following PICO (participants, interventions, comparisons and outcomes) question formed the basis for the systematic literature review: Among Pacific Islanders, does increased availability of and/or access to fish, compared to reduced availability or access, improve a) household food security and b) individual nutritional status? Depending on individual study designs, relative availability or access to fish was compared between communities or provinces within a single country, or between countries and/or the three Pacific sub-regions. In the case of studies conducted over different time periods, this was also analysed as change over time. For the purpose of this review, household food security is used in the context of either an individual within a household or a household comprising more than a single person. In relation to nutritional status, the influence of fish consumption on both under and over-nutrition was considered. Pacific Islanders were defined as individuals residing in the following PICTs: Commonwealth of the Northern Mariana Islands, Cook Islands, Federated States of Micronesia, Fiji, French Polynesia, Guam, Kiribati, Marshall Islands, Nauru, New Caledonia, Niue, Palau, Papua New Guinea, Pitcairn Island, Samoa, American Samoa, Solomon Islands, Tokelau, Tonga, Tuvalu, Vanuatu and Wallis and Futuna.

In December 2014, systematic searches were conducted using three major online academic literature databases. The Scopus database was searched using varying combinations of keywords, as listed in Table 2. Seven separate searches were conducted, with no new relevant articles being identified after search four. Additional searches were conducted in Web of Science and Medline databases using the same search strategy.

**Table 2:** Search terms used to identify articles for review

Keywords	
	“Pacific Island*” OR Kiribati OR Tuvalu OR Micronesia OR “Papua New Guinea” OR Nauru OR Palau OR “Solomon Island*” OR “Marshall Island*” OR Samoa OR “American Samoa” OR “Cook Island*” OR Fiji OR “New Caledonia” OR Tokelau OR “French Polynesia” OR Niue OR Tonga OR Guam OR Vanuatu OR “Pitcairn Island” OR “Wallis and Futuna” OR “Northern Mariana Island*”
AND	Fish*
OR	“Food securit*” OR “food insecurit*” OR “food suppl*”
OR	Nutrition OR “nutrition* status” OR “nutrition* outcomes” OR malnutrition OR “under nutrition” OR diet OR wasting OR stunting OR underweight

*denotes that any word preceded by this term could be included as a search term

Articles from the peer-reviewed literature in English published between 2004 and December 2014 were eligible for inclusion if they addressed either fish consumption amongst Pacific Islanders and/or the contribution of fishing activities to the livelihoods of Pacific Island people. Fish consumption could include either fresh or canned fish. Fishing activities could relate to both commercial and subsistence practices. Exclusion criteria were: review articles, studies focused on historical fish consumption in PICT communities, studies of institutionalised individuals, studies focused on the contribution of the fishing industry to a country’s national revenue or gross domestic product and studies describing fishing and fisheries, such as annual fish catch, that did not relate the activity to income generation or provision of subsistence.

As a broad range of search terms were needed to identify articles relevant to both nutrition and food security, the initial database search returned a large number of records. To remove subject areas irrelevant to the review, exclusions were made using database functions (Table 3). Records were exported to EndNote version X7 and duplicates removed. A title and abstract search was carried out to screen records against the inclusion and exclusion criteria. Eligibility of any remaining articles was determined through a full text assessment. Where there were aspects of doubt over a study’s eligibility, the investigator conducting the search (EG) sought a second and third opinion from other members of the research team. The review followed the PRISMA guidelines [32] for reporting systematic literature reviews.

**Table 3:** Subject areas excluded using database functions

Scopus	Web of Science	Medline
Neuroscience	Parasitology	Function not available
Psychology	Toxicology	
Computer science	Infectious diseases Pharmacology and pharmacy	
Dentistry		
Chemical engineering	Nuclear science technology	
Engineering	Clinical neurology Neurosciences	
Mathematics		
Material sciences	Surgery	
Physics and astronomy Pharmacology, toxicology and pharmaceutics	Medicine research experimental	
	Engineering chemical Virology	
	Computer science interdisciplinary applications	
	Radiology nuclear medicine medical imaging	
	Operations research management science	

Online resources, including the websites of the Food and Agricultural Organisation (FAO), the World Health Organisation (WHO), the World Bank, WorldFish, the Secretariat of the Pacific Community and the United Nations International Children’s Emergency Fund were also searched using the same approach to identify relevant grey literature.

Additionally, one country from each of the three cultural sub-regions within the Pacific Islands was examined in more detail to identify economic, demographic and health differences among sub-regions. These islands were Vanuatu in Melanesia, Kiribati in Micronesia and Tonga in Polynesia.

## Results

**Fig. 1:**
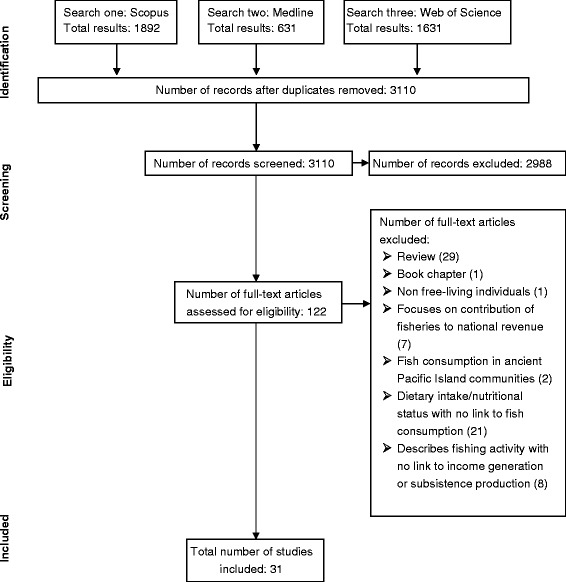
PRISMA flowchart of search process [32]

A total of 31 articles were identified for inclusion in the final review (Fig. 1). In two instances, two articles described results from the same study [33–36], and all four articles were included in this review. The findings are summarised, by country, according to two themes, fish consumption (shown in Table 4) (33–55, 57–59), and contribution of fishing to household income (shown in Table 5) (37, 42, 43, 45, 60-64). In addition, the three sub-regional case studies are described in Tables 6, 7 and 8. Only relevant papers identified using the defined search strategy are cited in the tables whereas additional sources of information are included in the Discussion.

**Table 4:** Characteristics and main findings of studies assessing fish consumption^a^

Reference	Population	Design^b^	Outcome measures^b^	Key findings^b^
Melanesia				
Fiji				
Hedges et al. [54]	20 non-pregnant non nursing females in Verata	Cross sectional study : a) Food records - food and beverages consumed over two x weekly periods, 3 weeks apart	Energy derived from protein, fat and carbohydrate (CHO), intake of protein and sources of protein intake	Mean energy from protein/carbohydrate/fat over the 2 weeks was 13 %/66 %/21 % respectively. Mean intake of energy was 9080 KJ/day. Mean intake of protein was 70.6 g. Primary sources of protein were cereals (3.7 % of protein energy) followed by fish (3.4 %), meat (1.7 %) and shell fish (1.5 %). Higher consumption of marine foods was associated with lower cereal intake (−0.54).
Kuster et al. [45]	Ono-i-Lau Island 30 senior heads of households in 1982 59 households in 2002	Cross sectional	Mean weekly household income and fish yield per capital. Daily fish intake per capita (g) and contribution of marine sources to protein intake.	Total annual landings of finfish decreased by 27 % from 1982 to 2002 No significant change in yield of finfish per capita of population (96.9 kg per capita/year in 1982 to 93.7 kg capita/year in 2002) Seafood remained the main source of protein between 1982 and 2002. Consumption of canned fish increased from 9 g/man/day to 19 g/man/day
Turner et al. [42]	Lau Province, 25 experienced local fishers 53 senior heads of households	Cross sectional Semi structured face-to-face interviews	Time spent fishing, importance of fishing for income generation, patterns of fish consumption and awareness of ecological change within the local qoliqoli (fishing ground)	No significant change in overall time spent fishing in the past 6–10 years. Income-generating activities had increased in importance over previous10 years relative to fishing activities Consumption of fresh fish was significantly lower compared to estimates of past consumption (Z = −3.774, *p* < 0.001). Greatest decline in fish consumption was associated with highest mean household income. Of the 80–100 % of households that engaged in fishing, only 7 % of households ranked fishing as the primary household occupation.
New Caledonia				
Guillemot et al. [44]	146 local fishers on the North west coast	Cross sectional study: Face-to-face questionnaire	Average number of fishing trips per year, average catch per fishing trip (kg) and annual reef fish consumption per capita	Total catches of reef fish estimated at 169 t/year. 7720 fishing trips made/year. Mean catch per fishing trip per boat was 23 kg. Annual reef fish consumption estimated at 18 kg/person/year
Labrosse et al. [50]	646 individuals aged over 7 years, in the Northern Province	Cross sectional study: Face-to-face 13-item questionnaire	Weekly and annual fish intake, quantity of fish per meal (g), subsistence versus purchased fish intake	Only 1.3 % of the participants reported never eating fish. 85 % of participants ate fish 1 or more times a week, with 45 % of these eating fish 2–3 times/week and 11 % consuming fish 1–2/day. Average weekly consumption of fish was 4.8 meals ± 0.7. Average quantity of fish consumed at a meal was 233 g ± 16 g. 92 % percent of annual fish consumption was classified as subsistence with only 8 % purchased.
Leopold et al. [48]	38 households on Ouvea Island	Cross sectional study: Face-to-face household questionnaire	Annual fish consumption per household and per consumption unit (CU)	Annual fish consumption per household was a mean 289 kg. Mean fish consumption per CU was 63 kg ± 9.7 kg (SD). Subsistence production was the main source of supply of fish for two thirds of households, followed by gifts and purchases from the market.
Solomon Islands				
Aswani & Furusawa [52]	Five villages in the Roviana Lagoon, 106 adult males and females 574 males and females aged ≥15 years 437 males and females ≥18 years (231 in 2001 and 206 in 2005)	Cross sectional Face-to-face semi-structured interviews, 24 h dietary recall Anthropometric assessment and 1 h dietary recall questionnaire	Contribution of marine resources to protein intake Energy (MJ) and protein (g) intake	64 – 100 % of all participants identified fish as the main source of protein consumed by their household Males and females consumed a sufficient amount of protein according to the FAO/WHO/UNU reference values 1985. Between 2001 and 2005 mean energy intake in both males (*P* < 0.01) and females (*P* < 0.0001) increased. There were no statistically significant changes in BMI for either males or females between 2001 and 2005.
Mertz et al. [41]	46 heads of households in Tikopia	Cross sectional Face-to-face household questionnaire	Fish and meat intake;	Fish consumed daily by 72 % of households. Main source of dietary protein was fish Imported canned fish and meat were eaten when available, but was rarely available.
Papua New Guinea				
van der Heijden [57]	609 individuals residing in high, middle and low altitude villages in the Ramu catchment and 204 fishers from the Yonki Reservoir (high altitude)	Cross sectional study: Structured face-to-face interviews	Contribution of fish to intake of animal based foods	Fish and other aquatic animals caught in nearby waters contributed 7.7 % of food from animal sources for high and middle altitude participants. Fish was the most important source of animal protein for low altitude respondents (41 % of all food from animal sources) and the second most important source for respondents at Yonki Reservoir (25 % of all food from animal sources). Canned fish and lamb flaps were the most commonly purchased animal foods
Vengiau et al. [34]	70 adult Naasioi migrants residing in Port Moresby aged 18–65 years	Cross sectional study: Face-to-face 39-item FFQ (foods were classified as ‘traditional foods’ or ‘store foods’) Assessment of household socio-economic status determined by fortnightly income, educational level, house type and number of household possessions	Association between socio-economic status and food intake type	“Socio-economic index” was positively correlated with greater consumption of “traditional foods” e.g., bananas, tubers, fruits and vegetables compared to “store foods” e.g., rice, noodles, canned fish and soft drink (*P* = 0.04)
Vengiau et al. [35]	70 adult Naasioi migrants residing in Port Moresby aged 18–65 years	Cross sectional study: Face-to-face 39-item FFQ to establish key dietary patterns Anthropometric assessment including height (cm), weight (kg), and blood pressure (BP)	Identification of key dietary patterns Association between dietary pattern and BMI (kg/m^2^) and blood pressure (mm Hg)	Two dietary patterns emerged. ‘Traditional diet’ - which was correlated with consumption of bananas, tubers, other sweet potato, pawpaw and leafy greens and negatively correlated with consumption of rice, canned fish and soft drinks. The second dietary pattern was correlated with consumption of store bought foods e.g., rice, noodles, canned fish and soft drinks Median BMI was 27 in females and 29 in males. 38 % of females and 23 % of males classified as obese (BMI ≥ 30). Systolic BP was > than 140 mm Hg in 8 % of females and 19 % of males Diastolic BP was > than 90 mm Hg in 10 % of females and 13 % of males. No association was found between cardiovascular risk factors (BMI and BP) and dietary patterns
Republic of Vanuatu				
Dancause et al. [46]	425 children and 559 adults on three islands varying in economic development (Ambae, Aneityum and Efate)	Cross sectional study: Face-to-face behavioural questionnaire 24 h dietary recall	Behavourial changes within and among islands associated with economic development	Hypertension or CVD among family members was reported by 19.7 % of participants in Ambae, by 35.3 % in Aneityum and 45.1 % in Efate. Overweight and obesity amongst family members was reported by 24.8 % of participants in Ambae, 48.0 % in Aneityum and 39.6 % in Efate. Fresh fish intake ranged from approximately 10 % in Ambae to 50 % in Aneityum. Meat and/or fish intake was highest in Efate, followed by Aneityum then Ambae (P < 0.001). Tinned meat was consumed only monthly or yearly by the majority of participants in Ambae and Aneityum, compared to consumption daily or weekly for 80 % of participants in Efate. Children in Efate reported taking more processed foods to school compared to children from Aneityum and Ambae who reported taking more local foods to school.
Dancause et al.[59]	534 males and females aged ≥18 years on three islands: Ambae, Aneityum and Efate	Cross sectional study: Face-to-face behavioural questionnaire 24 h dietary recall questionnaire Anthropometric assessment including height, weight (kg),	Association between fish and meat intake and anthropometric measures	For males and females means of anthropometric measures were lowest in Ambae (rural), intermediate in Aneityum (rural with tourism) and highest in Efate (suburban). Risk factors for obesity included consuming canned fish (OR 2.91, 95 % CI; *P* = 0.020) and consuming multiple fish/meat dishes (OR 3.24, 95 % CI; *P* = 0.010)
Li et al. [39]	153 school children aged 8–10 years from four locations on the island of Tanna	Cross sectional study: Face-to-face dietary survey Spot urine sample Thyroid volume	Intake of fish and type of fish consumed Median urinary iodine excretion (UIE), iodine sufficiency and association between fish consumption iodine sufficiency Association between thyroid volume and UIE	39 % of children reported eating fish on at least a weekly basis and 44 % on a monthly basis. Canned fish consumption was reported by 70 % of participants. Eating fish at least monthly was associated with urinary iodine sufficiency (UIE > 100 ug/L, *P* = 0.011; *P* = 0.045 after adjusting for location, age and sex). Children were moderately iodine deficient and had much larger thyroid glands compared to international reference values.A statistical significant inverse correlation between thyroid volume and UIE for boys (*r* = −0.444, *P* = 0.001; n 77) and girls (*r* = −0.319, *P* = 0.005; n 76)
Polynesia				
French Polynesia				
Clero et al. [36]	229 cases :203 women and 26 men diagnosed with thyroid cancer from 1979 to 2004 371 controls: matched to cases by sex and age	Case–control study: 66-item FFQ	Energy intake (kcal), iodine nutrition status and risk of thyroid cancer	Cases consumed a mean 71 g/day of fish and shellfish compared to 83 g/day in controls. Iodine nutrition deficiency (<150 ug/day) was observed in 60 % of cases and controls. 30 % had optimal iodine nutrition (150–299 ug/day). Risk of thyroid cancer decreased significantly with increasing consumption of fish (*P* = 0.008) and shellfish (*P* = 0.002). Subjects with a severe or moderate iodine nutrition deficiency had a 2.6 times increased risk of thyroid cancer (95 % CI: 1.12, 5.93) compared to subjects with optimal iodine nutrition status.
Clero et al*.*[33]	229 cases :203 women and 26 men diagnosed with thyroid cancer from 1979 to 2004 371 controls: matched to cases by sex and age	Case–control study: 66-item FFQ	Association between dietary pattern and thyroid cancer risk	2 major dietary patterns were identified: Western and traditional Polynesian. The traditional Polynesian pattern was inversely associated with risk of thyroid cancer. After adjustment for total energy intake, a 42 % reduced risk of thyroid cancer was found for the highest vs. the lowest tertile: (OR −0.58; 95 % CI: 0.35, 0.95; *P* = 0.02). The decreased risk was attenuated after multivariate adjustment (*p* = 0.2)
Dewailly et al.[38]	214 pregnant women who gave birth between October 2005 and February 2006 and their neonates	Cross sectional study: Face-to-face survey Blood sample taken from the umbilical cord of neonates	Monthly fish intake (meals/month) including type of fish Iodine (I), selenium (Se) and omega 3 polyunsaturated fatty acid (n-3 PUFA) concentrations	In pregnant women, mean fish consumption was 33 meals/month of which 21.3 and 11.5 meals/months were from reef and pelagic fish respectively. Tuna (75 %) was the most common pelagic fish species consumed. Mean umbilical iodine cord blood concentrations varied between different archipelagos. Highest mean concentration of iodine was 2.60 umol/L and the lowest was 0.46 umol/L. Mean cord blood selenium concentration was 2.0 umol/L. Mean concentration of n-3 PUFAs in red cell membrane phospholipids was 3.52 %. Eicosapentanoic acid (EPA) and docosahexanoic acid (DHA) represented 87 % of all n-3 PUFAs
Kingdom of Tonga				
Konishi et al*.* [53]	19 females (aged 40–59 years) and their spouses (15) from Kolovai village	Cross sectional study: Face-to-face 24 h dietary recall administered over 7 consecutive days during two different seasons Anthropometric assessment of weight and height	Protein intake (g) and contribution of local fish and imported meats to nutrient intake Weight (kg), height (cm) and BMI (kg/m^2^)	Daily protein intake was 112 g and 97 g for men and women respectively. 97 % of participants were considered to have adequate protein intake. Fish contributed 20 % toward total protein intake compared to the sum of the two major imported meats mutton and chicken (23 % of total protein intake). Mutton (23 %) and chicken (10 %) were the highest sources of fat in the diet. Mean BMI was 32.3 kg/m^2^ ± 4.4 kg/m^2^ and 36.3 kg/m^2^ ± 5.4 kg/m^2^for men and women respectively
Kronen & Bender [43]	Lofanga Island Individual adults (>15 years), households, key informants, 41 local fisherman	Mixed methods Households and individuals: structured questionnaires. Key informant interviews Fishermen: Semi-structured interviews	Fishing practices including contribution of fish to livelihood and weekly fish consumption through	Fisheries, agricultural production and handicrafts all contributed to income generation. Fisheries ranked higher than agriculture and handicrafts. Fisheries filled three main objectives: to secure subsistence, fulfil social obligations and contribute to the cash economy. Fish^a^ was consumed by entire community. 93 % of village also consume other seafood and 78 % consume canned fish.
Smith et al*.* [49]	443 school students aged 11–16 years from Tongatapu, Vava’u and Hapa’ai.	Cross sectional study: Self-administered health behaviour survey Anthropometric assessment including height (cms) and weight (kgs)	Intake of canned fish, mutton and beef Prevalence of overweight and obesity	35 % of participants reported consuming canned fish once or more a day compared to 57 % who reported consuming canned mutton or beef once or more a day The prevalence of overweight and obesity was 36 % amongst boys and 54 % amongst girls using international cut-off points for BMI.
Samoa and American Samoa				
Craig et al*.* [47]	594 fishermen and 20 males aged 43–72 years from Ofu, Olosega and Sili villages in American Samoa	Cross sectional Observation of fishing activities and semi-structured interviews with fishermen Free-form interviews of 20 males	Annual fish consumption	Per capita fish catch was 71 kg. 63 kg of catch was consumed per capita per annum. Remaining catch was either sold locally (60 % of fishers sold catch at least occasionally) or sent to family members on Tutuila Island.
DiBello et al*.* [58]	723 American Samoans and 785 Samoans aged ≥18 years	Cross sectional study: Anthropometric assessment (weight (kg), height (cm) and waist circumference). Fasting blood samples and blood pressure were measured. FFQ (42-item American Samoa and 55-item Samoa)	Prevalence of metabolic syndrome Association between dietary pattern and metabolic syndrome	The prevalence of metabolic syndrome was 49.4 % and 30.6 % in American Samoan and Samoan samples respectively ‘Neo traditional’ and ‘modern’ dietary patterns derived. The ‘neo-traditional’ pattern was characterised by high intake of crab and lobster, fish, coconut cream dishes, papaya soup, coconut milk, papaya.Significant increasing prevalence of metabolic syndrome across increasing quintiles of the ‘modern’ dietary pattern in Samoa (*P* = 0.05). The ‘neo-traditional’ dietary pattern was associated with an increase in HDL cholesterol in America Samoa (*P* = 0.02) and decreased waist circumference in both communities (P = 0.03).
Micronesia				
Federated States of Micronesia				
Corsi et al*.* [37]	293 females aged 15–64 years located in Ponhpei	Cross sectional study: 27-item, 7-day FFQ Knowledge, attitudes and practices questionnaire	Fish and meat consumption Cash expenditure on food; factors affecting food intake	79 % of participants reported frequent consumption of local fish/seafood. Local fish/seafood was consumed twice as frequently (4.8 days/week) compared to imported fish/seafood (2.4 days/week). Imported meats such as turkey tail were consumed more frequently 1.9 days/week) than local meats (1.3 days/week). 8 % of participants reported their household relied on farming and fishing for their primary income 6 % relied on fishing alone. Household food expenditure for 77 % of participants was half or more of their monthly income 52 % of participants purchased local food for half or more than half of a month. Consuming imported food was regarded as a sign of wealth and status by participants
Englberger et al*.*[55]	Kosrae 267 children aged 24–59 months and their caretakers 65 children aged 24–59 months and their caretakers	Cross sectional study: 34-item 7-day FFQ Three non-consecutive 24 h dietary recall questionnaires	Intake of total Vitamin A, retinol and protein (g). Sources of vitamin A	Main dietary components included imported products of rice, flour, chicken, other meats and tinned fish and local products of breadfruit, banana, taro, fish and other seafood. The most frequently consumed protein food was imported frozen chicken, followed by local fish. Mean intake of protein (54 g ± 10 g), well above the estimated requirements. Mean daily intake of total vitamin A for all children was less than half of the requirements estimated by WHO and the FAO^#^. Animal sources provided 52-53 % of vitamin A intake for both children and caretakers.
Guam				
Pobocik et al*.* [51]	211 males and 189 females	Cross sectional study: Multiple-pass telephone 24 h dietary recall Self reported weight (kg) and height (m)	Fish and other meat intake BMI (kg/m^2^)	Most commonly reported meats consumed were chicken (reported 159 times), beef (148), fish (141), eggs (75), sausage/bacon/hot dogs (65), pork/ham (60) and canned meat (42). Canned and processed meats accounted for 21 % of all reported meat intake. When fish was consumed 47 % was fresh and the remaining was canned or dried. Mean BMI was 25.7 kg/m^2^ ± 5.8 kg/m^2^ with significant differences in BMI by ethnicity (*P* < 0.05)
Multiple PICTs				
Phongsavan et al*.* [40]	4885 school children aged 13–15 years from Republic of Vanuatu, Kingdom of Tonga and Federated States of Micronesia	Cross sectional study: Self-administered Health Behaviour and Lifestyle of Pacific Youth (HBLPY) survey.	Canned fish and mutton intake	In Vanuatu, canned fish was consumed on a daily basis by 40 % of students and canned mutton 17 % of students. In Tonga 32 % of students consumed canned fish on a daily basis and 52 % of students consumed canned mutton daily. In Pohnpei students were asked about consumption of fresh fish - 42 % reporting consuming fresh fish on a daily basis and 46 % consumed canned mutton daily

^a^Fish: Refers to fresh fish unless otherwise specified. ^b^ Note: When describing the design, outcome measures and findings of each study only details relevant to this systematic literature review were included in the summary table. *BMI* body Mass Index, *FFQ* Food Frequency Questionnaire, *WHO* World Health Organisation, *FAO* Food and Agricultural Organisation of the United Nations

**Table 5:** Characteristics and main findings of studies assessing the contribution of fishing to Pacific Islander livelihoods

Reference	Study Population	Study Design^a^	Outcome measures^a^	Key findings^a^
Melanesia				
Fiji				
Kuster et al*.* [45]	Ono-i-Lau Island 30 senior heads of households in 1982 59 households in 2002	Cross sectional	Mean weekly household income and fish yield per capital. Daily fish intake per capita (g) and contribution of marine sources to protein intake.	Total annual landings of finfish decreased by 27 % from 1982 to 2002 No significant change in yield of finfish per capita of population (96.9 kg per capita/year in 1982 to 93.7 kg capita/year in 2002) Seafood remained the main source of protein between 1982 and 2002. Consumption of canned fish increased from 9 g/man/day to 19 g/man/day
Middlebrook & Williamson [61]	Ucunivanua and Namatakula, Island of Viti Levu 40 heads of households	Cross sectional Self administered household questionnaires	Household income, source of income and household fishing activity	Ucunivanua: Mean monthly household income of FJ$ 411.75 ± 73.51 (SD). Income generated through fishing activities (75 %), with 20 % from farming and 5 % from wage-paid jobs. Namatakula: Mean monthly household income of FJ$ 432.25 ± 54.65 (SD) a month. Income generated through wage-paid work (80 %), with 10 % from fishing and 10 % from personal business.
O’Garra [60]	Navakavu fishing grounds, Rewa, 118 heads of households 86 adult individuals (aged >21 years)	Cross sectional household and individual questionnaires	Socio-demographic characteristics of household, household livelihood activities and household fishing activities	Mean annual household income was FJ$ 2921. 88.1 % of households engaged in fishing, 76.3 % in growing crops and/or gleaning. The livelihood that generated the most cash and food overall was salaried work in Suva (32 % households) followed by fishing (27 %) and gleaning (20 %).
Turner et al*.* [42]	Lau Province, 25 experienced local fishers 53 senior heads of households	Cross sectional Semi structured face-to-face interviews	Time spent fishing, importance of fishing for income generation, patterns of fish consumption and awareness of ecological change within the local qoliqoli (fishing ground)	No significant change in overall time spent fishing in the past 6–10 years. Income-generating activities had increased in importance over previous10 years relative to fishing activities Consumption of fresh fish was significantly lower compared to estimates of past consumption (*Z* = −3.774, *p* < 0.001). Greatest decline in fish consumption was associated with highest mean household income. Of the 80–100 % of households that engaged in fishing, only 7 % of households ranked fishing as the primary household occupation.
Solomon Islands				
Albert et al*.* [64]	Western Province and Guadalcanal Province Households in four villages with Fishing Aggregating devices (FADs)	Cross sectional monitoring of fishing activities key informant interviews	Annual fish catch and contribution of FAD to fish catch Benefits and negative aspects of the FAD at the household and community level	Near shore FADs contributed 31–45 % of the total annual catch (mean 7500kgs). Perceived benefits from the FADs included: provided a source of family income, improved nutrition, more fish available for community events. The negative aspect of FADs was a reduced contribution of fishers to household activities due to increased time spent fishing
Papua New Guinea				
Cinner et al. [63]	Ahus Island 51 households representatives	Cross sectional Face-to-face interviews	Percent of households engaged in fishing and importance of fishing relative to other livelihood activities	>96 % of households were engaged in fishing and >76 % ranked fishing as their primary occupation. Due to the remoteness of the Island participants reported few opportunities to engage in other economic sectors.
Polynesia				
French Polynesia				
Walker & Robinson [62]	Moorea 70 females and males (aged 18–84 years)	Cross sectional interviews with open-ended, semi-structured and structured questions	Fishing activities including subsistence and commercial activities	60 % of participants fished on average 2 days per week; 10 % did not fish at all. 56 % of participants reported dependence on lagoon fishing for at least half of their food and/or cash income. 19 % ofparticipants considered themselves commercial fishers and 41 % subsistence fishers
Kingdom of Tonga				
Kronen & Bender [43]	Lofanga Island Individual adults (>15 years), households, key informants, 41 local fisherman	Mixed methods Households and individuals: structured questionnaires. Key informant interviews Fishermen: Semi-structured interviews	a) Fishing practices including contribution of fish to livelihood and weekly fish consumption through	Fisheries, agricultural production and handicrafts all contributed to income generation. Fisheries ranked higher than agriculture and handicrafts. Fisheries filled three main objectives: to secure subsistence, fulfil social obligations and contribute to the cash economy. Fish^b^was consumed by entire community. 93 % of village also consume other seafood and 78 % consume canned fish.
Micronesia				
Federated States of Micronesia				
Corsi et al*.* [37]	293 females aged 15–64 years located in Ponhpei	Cross sectional study: 27-item, 7-day FFQ Knowledge, attitudes and practices questionnaire	Fish and meat consumption Cash expenditure on food; factors affecting food intake	79 % of participants reported frequent consumption of local fish/seafood. Local fish/seafood was consumed twice as frequently (4.8 days/week) compared to imported fish/seafood (2.4 days/week). Imported meats such as turkey tail were consumed more frequently 1.9 days/week) than local meats (1.3 days/week).8 % of participants reported their household relied on farming and fishing for their primary income 6 % relied on fishing alone. Household food expenditure for 77 % of participants was half or more of their monthly income 52 % of participants purchased local food for half or more than half of a month. Consuming imported food was regarded as a sign of wealth and status by participants

^a^Note: When describing the design, outcome measures and findings of each study only details relevant to this systematic literature review were included in the summary table ^b^Fish: Refers to fresh fish unless otherwise specified

**Table 6:** Vanuatu case study: demographic, nutrition and fish data

Indicator					Result	Ref
Population	All				276, 579	[94]
	<5 years				35 000	[95]
Life expectancy at birth	Male				70 years	[96]
	Female				73 years	
Births per year					7911	[2]
Mortality/1000	Infant				28	[96]
	Under 5				31	
Household expenditure on food					49.8 %	[97]
Nutrition						
Vitamin A deficiency in pre-school aged children					16 %	[98]
Nutritional status among children <5 years:	Stunting				28.5 %	[96]
	Wasting				4.4 %	
	Underweight				10.7 %	
Nutritional status among adults	overweight or obese:		Women		49.5 %	[96]
aged 15–49 years			Men		35.8 %	
	Raised blood pressure:				47.2 %	[99]
	Raised blood glucose:				19 %	[100]
	Raised blood cholesterol:				37.6 %	[101]
Energy (kcal) available per capita					2820	[102]
Diet composition of population:	Carbohydrate				60 %	[103]
	Fat				31 %	
	Total protein				9 %	
Infant feeding practices:	Children < 5 years are ever breastfed				94.9 %	[96]
	Infants exclusively breastfeed to 5 months				72.6 %	
Households consuming adequate iodised salt:					50.7 %	[96]
Fish and fisheries	National Urban		Rural	Coastal		[27, 97]
Annual per capita fish consumption (kg)	20.3	19.3	20.6	29.9		
Subsistence fishing	51 %	17 %	60 %	-		
Purchased	49 %	83 %	40 %	-		
Consumption of fresh fish	60 %	38 %	65 %	72 %		
Fish as % of total protein consumption	Total protein				14.9 %	[102]
	Animal protein				35.8 %	
Forecasts of fish required for food (tonnes)	2010				8200	[27]
	2020				10 700	
	2030				13 600	
Rural households engaged in fishing activities					72 %	[104]
Dependence on fisheries for income :	Marine fishing activities			Urban	11 %	[105]
				Rural	39 %	
	Fresh water fishing activities			Urban	4 %	
				Rural	21 %	
Official fishing contribution to GDP					0.8 %	[104]
Contribution of fish access fees to national revenue					1.2 %	[104]

**Table 7:** Kiribati case study: demographic, nutrition and fish indicators

Indicator					Result	Ref
Population	All				101,000	[95]
	<5 years				11 000	[95]
Life expectancy at birth	Male				65 years	[106]
	Female				70 years	
Births per year					2000	[107]
Mortality/1000	Infant				25.6	[108]
	Under 5					
					60	
Household expenditure on food					46 %	[109]
Nutrition						
Vitamin A deficiency in pre-school aged children					22 %	[98]
Nutritional status among children <5 years:	Stunting				--	
	Wasting				--	
	Underweight				14.9 %	[108]
Nutritional status among adults	overweight or obese:		Women		78.9 %	[110]
aged 15–49 years			Men		67.4 %	
	Raised blood pressure:				37.4 %	[99]
	Raised blood glucose:				21.4 %	[100]
	Raised blood cholesterol:				35.5 %	[101]
Energy (kcal) available per capita					3022	[111]
Diet composition of population:	Carbohydrate				58.6 %	[103]
	Fat				31 %	
	Total protein				10.5 %	
Infant feeding practices:	Children < 5 years are ever breastfed				83 %	[108]
	Infants exclusively breastfeed to 5 months				69 %	
Households consuming adequate iodised salt:					5 %	[112]
Fish and fisheries	National	Urban	Rural	Coastal		[27]
Annual per capita fish consumption (kg)	62.2	67.3	58	115.3		
Subsistence fishing	63 %	46 %	79 %	-		
Purchased	37 %	54 %	21 %	-		
Consumption of fresh fish	92 %	91 %	93 %	95 %		
Fish as % of total protein consumption	Total protein				28.8 %	[29]
	Animal protein				55.9 %	
Forecasts of fish required for food (tonnes)	2010				7730	[27]
	2020				9050	
	2030				10 230	
Rural households engaged in fishing activities					60 %	[113]
Dependence on fisheries for income :	Sales of fish and agricultural			Urban	26 %	[113]
	crops for cash income:			Rural	34 %	
	Paid workers in the ‘agriculture				19.5 %	[113]
	And fisheries’ sector					
Official fishing contribution to GDP					0.8 %	[104]
Contribution of fish access fees to national revenue					42 %	[104]

**Table 8:** Tonga case study: demographic, nutrition and fish indicators

Indicator					Result	Ref
Population	All				103,252	[114]
	<5 years				14,000	[95]
Life expectancy at birth	Male				65 years	[115]
	Female				69 years	
Births per year					2,896	[114]
Mortality/1000	Infant				7	[116]
	Under 5				18	[116]
Household expenditure on food					50.6 %	[117]
Nutrition						
Vitamin A deficiency in pre-school aged children					17 %	[98]
Nutritional status among children <5 years:	Stunting				8.1 %	[116]
	Wasting				5.2 %	
	Underweight				1.8 %	
Nutritional status among adults	overweight or obese:		Women		79.6 %	[110]
aged 15–49 years			Men		69.9 %	
	Raised blood pressure:				41 %	[99]
	Raised blood glucose:				18 %	[100]
	Raised blood cholesterol:				50 %	[101]
Energy (kcal) available per capita					--	
Diet composition of population:	Carbohydrate				--	
	Fat				--	
	Total protein				--	
Infant feeding practices:	Children < 5 years are ever breastfed				91 %	[116]
	Infants exclusively breastfeed to 5 months				52.2 %	
Households consuming adequate iodised salt:					--	
Fish and fisheries	National	Urban	Rural	Coastal		[27, 117]
Annual per capita fish consumption (kg)	20.3	--	--	84.6		
Subsistence fishing	37 %	---	--	-		
Purchased	63 %	--	--	-		
Consumption of fresh fish	80 %	--	--	87 %		
Fish as % of total protein consumption	Total protein				13.5 %	[118]
	Animal protein				23.4 %	
Forecasts of fish required for food (tonnes)	2010				3,490	[27]
	2020				3,690	
	2030				3,900	
Households engaged in fishing activities					33 %	[119]
Dependence on fisheries for income :	Workforce employed in fishing industry				3 %	[120]
Official fishing contribution to GDP					4.1 %	[104]
Contribution of fish access fees to national revenue					0.2 %	[104]

### Fish consumption

#### Fish intake in Pacific Islanders

Fish was identified as a primary food source for Pacific Islanders in 15 studies [36–50] although the amount and type of fish consumed varied based on factors such as geographical location and socio-economic status. The following section describes the patterns in Pacific Island fish intake reported in these studies.

#### American Samoa

A study of subsistence fishing practices in two outer islands of American Samoa found that 89 % of the fish harvest was consumed locally, with the remainder being sent to family members on other islands. Interviews with local elders suggested that fishing activities had not changed much over their lifetime and that there were ample fish resources still available [47].

#### Federated States of Micronesia

In Pohnpei, an island in the Federated States of Micronesia (FSM), 79 % of adults reported frequent consumption of fresh fish and/or seafood with locally-caught fresh fish and/or seafood consumed 4.8 days/week compared to 2.4 days/week for imported fish and/or seafood, including canned fish [37]. This contrasts to results from the Health Behaviour and Lifestyle of Pacific Youth study (HBLPY), which reported that fresh fish was consumed by only 42 % of 15-year-old students at seven schools in Pohnpei, whereas there was high consumption of processed foods such as canned meat and carbonated beverages [40].

#### Fiji

Fish intake in Fiji was assessed by Kuster et al.[45] and Turner et al. [42]. In a comparison of subsistence fishing patterns between 1982 and 2002 on Ono-i-Lau Island, Kuster et al.[45] found that fresh fish remained the main source of dietary protein (finfish 261 g ± 90/person/day in 1982 and 269 g ±100/person/day in 2002) for villagers over a 20 year time span. Consumption of tinned fish doubled from 9 g per person per day to 19 g per person per day over the same period. In the remote Lau islands, local fishers and senior heads of households reported the frequency of consuming fresh fish had declined over the previous 10 years to an average current consumption level of three days per week. The decline in frequency of fish consumption was associated with higher household income. In Naikeleyaga, the Lau island village with the highest annual household income, participants reported the frequency of fish consumption declined from four to 1.5 days per week. Reasons identified for declining fish consumption were a dependence on other family members to catch fish, or a need to purchase rather than catch their own fish (possibly due to more time spent on income-generating activities [42]), as well as increased consumption of other purchased foods.

#### French Polynesia

Two separate studies assessed fish intake in French Polynesia. In a cross sectional study of pregnant women participants, there was a mean consumption of 33 meals/month of reef and pelagic fish, however, data on actual grams per day were not collected [38]. In a case–control study of thyroid cancer patients, an average of 71 g/day and 83 g/day of fresh fish and/or shellfish was consumed by cases and controls, respectively [36].

#### Guam

In contrast to other Pacific Islands, Pobocik et al. [51] reported that in adults from Guam, fish contributed only 3.5 % of foods eaten but was the third most commonly consumed meat, after chicken and beef. Of the fish that was consumed, only 47 % was fresh and the remaining canned or dried [51].

#### New Caledonia

Three studies assessed fish intake in New Caledonia. In the Northern Province, 85 % of adults participating in a study of subsistence fishing practices reported consuming fresh fish one or more times a week, with 45 % consuming fresh fish 2–3 times/week and 11 % 1–2 times/day [50]. Of annual fish consumption, 92 % was classified as subsistence and 8 % was purchased [50]. Subsistence production was also the main fish supply for two-thirds of households on Ouvéa Island, New Caledonia, with annual intake of fish considerably higher than reported in the Northern Province [48]. In a small study of 146 local fishers from the northwest coast, approximately 18 kg of fish per capita per annum was consumed, however, only reef fish intake was considered, not pelagic species nor imported fish [44].

#### Solomon Islands

In Tikopia, an isolated island in Solomon Islands, Mertz et al. [41] reported that 72 % of households consumed fresh fish daily, whereas canned fish was eaten only when rarely available [41].

#### Tonga

On Lofanga Island, Tonga, the entire community reported eating fresh fish, with 93 % also consuming other seafood and 78 % of the community consuming canned fish. However, a decline in fresh fish consumption was attributed to increased income resources [43]. In a cluster random sample of schools in Tonga, approximately a third (27.2 to 38.6 %) of 13- and 15-year-old girls and boys who completed the HBLPY study reported consuming canned fish on a daily basis and half the students consumed canned mutton daily [40]. However, it is not known how many students consumed fresh fish as those data were not collected.

#### Vanuatu

In Vanuatu, fish consumption varied between islands but, overall, fresh fish consumption was lower than for Solomon Islands and Tonga. The percentage of adults consuming fresh fish during the previous 24 h ranged from 10 to 50 %, depending on the island. No data were available on the amount of fish consumed [46]. On Aneityum, considered to be a rural island with tourism, children were more likely to consume tinned fish than adults. These findings were similar to those of Phongsavan et al. [40] who reported that 40 % of students in the HBLPY survey reported consuming canned fish on a daily basis. On the rural island of Tanna, 39 % of primary school children consumed fish at least weekly, with the majority (70 %) consuming mainly canned fish. Fish consumption differed among the four study sites in Vanuatu, with more fish consumed in coastal villages than in urban centres [39].

#### Contribution of fish consumption to energy and/or protein intake

Fish/seafood was also identified as the primary source of dietary protein in five of the six small-scale studies that addressed contribution of fish consumption to protein intake [41, 45, 52–54]. Consumption differed across PICTs, ranging from 20 % of total protein intake in the Kolovai village in Tonga [53] to 37.4 % in Verata, Fiji [54].

#### Federated States of Micronesia

In Kosrae, a cross-sectional survey of children and their caretakers reported that local fish was the second most commonly consumed source of protein, after cooked chicken [55]. Mean intake of protein, both for children and adults, was above the average requirements, as defined by Latham [56].

#### Fiji

In one rural village in Fiji, the second and third sources of protein intake after fish and shellfish (37 %) were cereals (29.2 %) and meat (13 %). Higher marine food consumption was associated with lower cereal intake [54].

#### Papua New Guinea

Fish consumption in the Sepik-Ramu catchment area in Papua New Guinea differed between the four villages surveyed (two high altitude, one middle and one low) [57]. In the low altitude village, fish was the most important source of protein for residents and comprised 41 % of all food from animal sources. Conversely, in high and middle altitude villages, animal protein comprised mostly purchased sources such as canned fish and lamb flaps [57].

#### Solomon Islands

In Solomon Islands, a cross sectional study of adults from five villages in Roviana Lagoon found that most households consumed fish as the primary source of protein but did not give details of alternative protein sources [52]. Mertz et al. [41] also reported fish as the primary source of protein among residents of Tikopia, a remote area in Solomon Islands. Just over half of households interviewed owned chickens, but meat from livestock was rarely eaten. Other sources of protein included canned fish and canned meat but these were rarely available.

#### Tonga

In Tonga, protein intake from fish was highest (20 %), followed by imported chicken (12 %) and mutton (11 %) [53].

#### Fish consumption and nutritional outcomes

Iodine status was reported in three studies [36, 38, 39]. In a case–control study of French Polynesians, inadequate iodine intake (<150 ug/day) was observed in 60 % of participants, with optimal iodine intake (150–299 ug/day) reported in only 30 % of participants. Subjects with thyroid cancer were 2.5 times more likely to have intakes of <75 µg/day compared to control subjects [36]. The iodine concentration of umbilical cord blood of newborn babies also suggested that iodine intake was adequate in French Polynesian mothers [38]. Fish intake of the women was assessed during pregnancy, with mean fish consumption reported as 33 meals per month (21.3 meals per month from reef fish and 11.5 meals per month from pelagic fish); fish intake varied little between women from different archipelagos. This suggests that fish was consumed daily, however, data on actual grams consumed per day were not collected [38]. Mean blood concentration of iodine varied between newborn babies from different archipelagos, with the highest mean concentration in the Iles Sous Le Vent (2.6 μmol/L) and the lowest in the Australes (0.46 μmol/L) [38].

Li et al. [39] assessed the iodine status of children on the Island of Tanna, Vanuatu, by measuring the level of urinary iodine excretion (UIE), and reported moderate population-level iodine deficiency (median UIE = 49 μg/l). Only 30 % of participants reported consuming mostly fresh fish, with the remainder eating mainly canned fish.

In Kosrae, Micronesia, Englberger et al. [55] reported that the mean daily intake of total vitamin A for all infants was less than half of the requirements estimated by the WHO and the FAO [55]. Yet both protein and fat intakes were above the estimated requirements and results were similar for infants’ caretakers. Highest intakes of total vitamin A and retinol were found in Malem, one of four Kosrae municipalities. Informants in the ethnographic analysis suggested that a wider range of seafood was consumed in Malem, including whole small fish consumed with the liver which is a rich source of vitamin A [55].

Assessing whole-of-diet against health outcomes was the approach used in five studies. A ‘traditional’ Polynesian dietary pattern, compared to a ‘modern’ dietary pattern, was shown to be associated with decreased risk of thyroid cancer in French Polynesia but the findings were no longer significant after accounting for several confounding variables including family history of thyroid cancer [33]. In Samoa, the ‘modern’ dietary pattern, characterised by high intakes of processed foods, was significantly associated with an increased prevalence of metabolic syndrome [58].

In Port Moresby, Papua New Guinea, two predominant dietary patterns were identified amongst a Naasioi migrant population [35]. Neither dietary pattern included high intakes of fresh fish but the ‘traditional’ dietary pattern was inversely associated with consumption of canned fish, rice and soft drinks [35]. Dietary patterns were not associated with body mass index (BMI) nor blood pressure [35]. A study of three villages in Vanuatu reported that both consumption of canned fish and multiple fish/meat dishes per day were risk factors for obesity, defined by both BMI and percentage body fat. Canned fish could lead to obesity because it is most commonly packaged with oil or sauce providing a higher fat content than fresh fish. Again, fresh fish intake was not considered in the analysis [59].

### Contribution of fishing activities to subsistence and/or cash income amongst Pacific Islanders

The degree to which individuals and households depended upon fishing for livelihood varied between and within PICTs. Four of the studies reviewed were conducted in Fiji, one of the more economically developed countries in the region [42, 45, 60, 61].

#### Federated States of Micronesia

Fishing as the primary source of income or food has been described in many PICTs however, this is not the case for all PICTs. Pohnpei, in the Federated States of Micronesia (FSM), is considered to be one of the urban centres currently experiencing the nutrition transition. It was found that 65 % of participants surveyed reported that one or more members of their household held a salaried position. Of the monthly household income, 77 % participants reported that household food expenditure made up half or more of the budget. In contrast, farming and fishing was the primary source of income in 8 % of participants and only 6 % relied on fishing alone [37].

#### Fiji

In the Navakavu fishing grounds in the south eastern area of Fiji, 88 % of households were involved in fishing activities. However, salaried work provided the most income and food for 32 % of households, while 27 % of households reported fishing as their most important resource [60]. On the island of Vitu Levu, two contrasting villages were studied; firstly, Ucunivanua, where fishing is the primary income activity for 75 % of households. In Namatakula, the primary income source was provided from employment in local international hotels (fishing being primary income for only 10 % of households) [61]. Despite primary sources of income differing between the two villages, there was no significant difference in total monthly income.

Key informants, local fisherman and senior heads of households, from four islands in the Lau Province of Fiji identified a decreasing dependency on fishing activities for livelihood over the past decade. Current fishing activities continued primarily for subsistence purposes, with the exception of fishing activities on Yaroi where fish was more commonly sold. Only 7 % of households ranked fishing as the primary household occupation although almost all households remained engaged in fishing practices [42]. In the most southerly Fijian Island group of Ono-i-Lau, also part of the Lau province, the economy was again predominantly subsistence and this had not changed between the two study periods of 1982 and 2002 [45]. While it was reported that time spent fishing had not significantly changed in the past 10 years, engagement in other forms of livelihood activities had increased including formal employment and animal husbandry [42]. Fishing and gardens were the primary source of subsistence products but the key income-generating activities were sale of copra, seaweed farming and handicraft making. Income from these sources doubled between 1982 and 2002, but income remained insufficient at both time points to purchase sources of protein other than fish on a regular basis [45].

#### French Polynesia

In Moorea, French Polynesia, a subsistence economy of small-scale agriculture and fishing existed until about 1962 [62]. In a small sample of adults interviewed in 2002, over half reported dependence on lagoon fishing for at least half of their food and/or income [62]. However, there has been an influx of tourism, and agricultural production for export markets, which have largely replaced local food production, including fishing.

#### Papua New Guinea

On the island of Ahus, Cinner et al. [63] found that more than 76 % of participants ranked fishing as their primary occupation. Participants reported that the remoteness of the island meant that there were few opportunities to engage in other economic sectors such as agriculture. Maintenance of marine tenure rights was important to create a demand for fish from the mainland [63].

#### Solomon Islands

The introduction of nearshore fishing aggregating devices (FADs) improved catch in four villages in Solomon Islands. These FADs contributed 31–45 % to the total annual catch and 26–58 % of the fish consumed annually [62, 64, 65]. The perceived benefits of FADs included provision of income through the sale of catch and improvements to diet through increased fish consumption [64].

#### Tonga

The Island of Lofanga is a traditional Tongan community in which the cash economy remains limited. On this island, fisheries, ranked higher than agriculture and handicrafts for importance to income generation, and were considered vital to secure subsistence and fulfil social obligations. However, in 45 % of households studied, income was also received as financial remittances from relatives on other islands or overseas [43].

### Country case studies

To highlight the heterogeneous nature of Pacific Islands, three PICTs, one from each of the cultural sub-regions, were examined in further detail. Demographic, nutritional and fish indicators from Vanuatu, Kiribati and Tonga (Tables 6, 7, and 8) demonstrate that fish consumption, as a percentage of total protein intake, varies considerably across the three cultural sub-regions. Fish consumption is much lower in Vanuatu, than in Kiribati and Tonga. This is presumably due to the relatively large population of Vanuatu and the limited area of coral reef capable of supporting coastal fisheries, resulting in low availability of fish per capita. The availability of beef cattle on some islands in Vanuatu, and the great importance of yams in the traditional diet, presumably also affect the role of fish in the nation’s food system.

Nevertheless, the patterns of fish consumption evident from the case studies summarised in Tables 6, 7 and 8 are representative of differences among the three sub-regions in general, with average national fish consumption in Micronesia and Polynesia, being much greater than in Melanesia [27]. The case studies support the data presented in the numerous individual country studies identified in this review and are used to present a succinct summary overview of factors influencing fish consumption patterns, food security and health status between the sub-regions.

## Discussion

This review confirms that fish is an important staple food in most Pacific Island countries and territories, and that subsistence and commercial fishing activities make essential contributions to both household and individual food security, particularly in rural areas. Estimates of per capita annual fish consumption ranged from 18 to 63 kg. The wide range of fish consumption can be attributed to variables such as geographical location (rural coastal villages versus urban centres), availability of alternative animal food sources (from both agriculture and imported foods) and whether the community has a subsistence or cash-based economy. These differences are highlighted in the three country case studies, showing higher fish consumption in Kiribati, one of the least developed PICTs.

Given the central contribution of fish to both dietary intakes and livelihoods in PICTs, it is noteworthy that our review identified only 29 relevant studies from 22 countries. Nationally representative data is sparse, and whole provinces and/or cultural groups are missing from the Melanesian countries. In a broader study, Bell et al. [27] used data from household income and expenditure surveys to quantify average national, rural and urban fish consumption per capita for 16 PICTs. Average national fish consumption ranged from 55 kg to 110 kg per person per year in eight PICTs, 3–6 times the average global consumption of ~18 kg per person per year [66]. Importantly, fish provided 50–90 % of dietary animal protein in rural areas across a wide range of PICTs.

With ongoing population growth, coastal fisheries based on coral reef ecosystems are not expected to yield sufficient fish to maintain per capita fish consumption in several PICTs [27]. The rich tuna resources of the region will need to play a greater role in maintaining the food security of Pacific Island people [31]. However, as urbanisation increases and urban centres become more focused on cash economies, the risk is that greater availability of imported, energy-dense, nutrient-poor foods will exacerbate the dual burden of malnutrition that includes micronutrient deficiencies and infectious diseases, accompanied by non-communicable diseases. Food security involves not only access to sufficient food, but also to nutritious food. Therefore, easy and affordable access to fish will be essential in order to provide growing PICT populations with healthy sources of food.

It has been estimated that for optimal nutrition, Pacific Islanders need to consume approximately 34–37 kg of fish per annum (based on WHO recommendation of 0.7 g protein/kg body weight/day and an assumption that fish will need to supply 50 % of required protein) [27]. Dependence on fish for protein is particularly high in rural areas where there is limited access to other sources of protein, such as imported meats, and in areas where animal grazing and husbandry is not viable [27, 67]. Our review suggests that the majority of PICT communities for which data is presented consume sufficient protein to meet their nutritional requirements, however, a number of communities, particularly inland PNG, suffer from high rates of protein-energy malnutrition [68, 69]. For these communities, an increase in the availability of fish has particular potential to contribute to improved nutritional outcomes. However, the remote nature of communities in PNG poses many challenges to increasing access to fish [27, 31]. To sustain recommended levels of fish consumption across the region as populations grow, new strategies are required to assist communities to obtain fish [27, 31]. A recent paper published after completion of this review reported that fishing remained the most important livelihood for households in the Langalanga region of the Solomon Islands but that households were involved in a range of different livelihoods in order to produce food and/or generate income. The authors suggested that households in this region had the ability to adapt to changing circumstances but that fisheries management involving local stakeholders was needed in order to ensure that fisheries continue to be a major source of food and income[70].

This review did not identify any reported interventions designed to test the hypothesis that increased availability of fish improves nutritional outcomes. Thus, our PICO question could not be answered directly. However, it is evident that fish is a highly nutritious food and that it provides a lean source of high biological value dietary protein as well as many essential fatty acids and micronutrients. In particular, the high iodine levels in fish and other seafood mean that improved access to increased fish availability may help prevent iodine deficiency, which remains highly prevalent in PICTs such as Fiji [71], PNG [72] and Vanuatu [39]. Improving iodine nutrition status through higher fish consumption may also contribute to the prevention of iodine-related diseases including goitre [39] and thyroid cancer [33, 36, 73]. In addition, the review suggests that some species of small fish that are eaten whole are important for the prevention of Vitamin A deficiency [30], which is highly prevalent in several PICTs including FSM [74], Kiribati [75], Marshall Islands [76], and PNG [72].

Sources of oily fish provide docosahexanoeic acid, and consumption of these fish by Pacific Islanders [38] may be making an important contribution to cognitive development in utero (through maternal fish intake), as well as in young children [30]. In other developing countries, such as Cambodia and Bangladesh, fish is an important source of calcium, iron and zinc [30], but data on the contribution of these nutrients from fish in PICTs are not available. Overall, further research is required to determine the extent of other micronutrient deficiencies that may exist in PICTs, such as iron or calcium and how fish consumption may contribute to reducing the risk of such deficiencies.

### Prevention and management of NCDs

Non-communicable diseases have been declared a crisis in the Pacific, and there are national response plans in place to address risk factors, particularly with regard to strategies to reduce population-level salt intake. Dietary survey work in Fiji identified the main sources of salt in the diet as salt (and MSG) added during cooking and at the table, as well as processed foods such as tinned fish and meat, biscuits, soy sauce, noodles, salty crisps and snacks, tinned fruit and vegetables, butter and bread [77]. Country consultations and Food Frequency Questionnaires (FFQ) carried out in Fiji, Samoa, Kiribati and Cook Islands, have confirmed this [78]. Locally salted, preserved fish and meat, and meals eaten in and out of the home, such as Chinese dishes, soups and curries are also a common part of the diet in the region.

An increase in the availability of fresh fish offers an alternative to foods with a high salt content. Increasing access to fresh fish also offers an alternative to imported meats, many of which are energy-dense and high in saturated fat (e.g., frozen chicken, canned mutton, turkey tails and sausages). These energy-dense foods have been associated with the prevalence of chronic diseases and over-nutrition in PICTs, and their inclusion in emerging dietary patterns confirms this, as identified by studies identified in the current review.

‘Modern’ dietary patterns, characterised by high consumption of foods such as potato chips, cake, rice, instant noodles, soup, and low intakes of local foods, have been shown to be associated with an increased prevalence of metabolic syndrome [58]. In contrast, ‘traditional’ Pacific Island dietary patterns high in fresh fish and seafood, as well as other local foods, such as coconut-based dishes, taro and papaya, have been associated with reduced prevalence of metabolic syndrome, increased HDL cholesterol and reduced waist circumference [58]. Heavy reliance on poor-quality imported foods [79] is exacerbating the genetic predisposition of people in FSM to obesity [80].

Whether fish is consumed fresh or canned needs to be considered. Findings have been inconsistent as to whether consumption of canned fish *per se* is a risk factor for obesity in PICTs. Data collected between 2001 and 2002 from rural Tongan communities reported that neither fresh fish nor canned fish were related to obesity [81]. However, the more recent finding of Dancause et al. [59] that consumption of canned fish was a risk factor for obesity in Vanuatu warrants further consideration, in the context of the nutrition transition [5]. Methodological limitations to dietary assessment should be noted; Dancause et al. used a single 24 h recall, which may not be appropriate to determine usual intake. Further, a higher consumption of tinned fish may simply be a proxy measure for a greater reliance on processed foods in place of fresh fish, as populations become more urbanized and adopt more westernised eating patterns. Both the nutrient content and the preparation methods of tinned fish may also contribute to its association with obesity. Tinned fish canned in oil or sauce has higher fat content than most types of fresh fish [82] and will often be served with instant noodles and rice [59]. Packaged instant noodles are popular in Vanuatu [83] and across most PICTs, and their inclusion in the diet has been shown to be associated with increased risk of obesity. In comparison, fresh fish and meat more often accompany dishes prepared with traditional root crops and vegetables, which are less energy-dense. A heavy reliance on tinned fish in urban areas was reported during the first nutrition survey conducted in Vanuatu as long ago as 1951 [84] and has since been observed in many other PICTs [83, 85, 86]. The Vanuatu Ministry of Health NCD survey in 1998 reported associations among obesity and daily consumption of non-traditional fat sources (OR = 2.19), including oil, margarine/butter, milk, fresh meat, poultry, tinned meat, and tinned fish [87].

In short, it appears that any association of canned fish with obesity and NCDs may well be related to the foods eaten with canned fish, as well as the added oil or sauce used to preserve fish when canned [59]. On its own, canned fish is a valuable alternative source of protein, when fresh fish is in short supply due to unfavourable fishing conditions. While fresh fish remains a major part of the diet in Vanuatu, it is not available in all areas and only seasonally in others as shown in Table 6. It has been proposed, somewhat controversially, by Dancause et al. [59] that fresh meat might be a better dietary option than tinned fish in some PICTs, at least from the point of view of preventing NCDs. This requires further investigation with well designed intervention studies, but is considered impractical due to the limited scope for grazing cattle in most PICTs, as well as lack of refrigeration for storage. Animal husbandry (chickens and pigs) is already practiced widely but may have limited potential for further expansion due to the high costs of imported feeds.

The challenge is to encourage Pacific Islanders to maintain traditional dietary patterns in the face of urbanisation accompanied by the nutrition transition. To date, there have been surprisingly few publications on interventions in this regard. In FSM, a health promotion programme that encouraged the consumption of local foods, including banana, taro and breadfruit, demonstrated increased dietary diversity through higher consumption of local foods and a decrease in consumption of imported foods (such as rice) [91]. Such efforts could be used to promote the consumption of fresh fish in areas where intake of imported meats has become increasing prevalent.

### Food security

Fishing is critical to household food security in PICTs through subsistence production and income generation (which allows food purchases) especially for communities that have limited opportunity to engage in other agricultural or economic sectors to produce food and/or income [63, 88]. For example, although Fiji is one of the more developed PICTs, both subsistence and commercial fisheries remain an important source of food and livelihoods [60].

In 2010, at the Pacific Food Summit, a Framework for Action on Food Security in the Pacific was developed with four goals aligned to the WHO’s four pillars for food security. The first goal, availability of food, has been addressed to some extent in the literature, with predictions that the majority of PICTs will have sufficient fish for local consumption and commercial activities, as long as Pacific Island governments provide greater access to regional tuna resources and the bycatch from industrial fishing operations for local consumption [31]. Even so, inter-annual variation in where bycatch is landed by fishing fleets could cause temporary shortfalls in fish supply in some urban centres [88].

Strategies such as increasing nearshore FADs have shown promise in terms of how increased availability of tuna and other large pelagic fish can help provide the protein that communities need for food security and nutrition [65], however appropriate monitoring and management is required. Strategies based on facilitating the supply of locally-canned tuna to the large inland population of PNG have yet to be developed in any detail but are much needed [27, 31].

The overview study by Bell et al. [27] showed that ~50–90 % of fish consumed in rural areas of PICTs was obtained through subsistence fishing. Other research done by the Coastal Fisheries Programme at the Secretariat of the Pacific Community [89] shows that an average of ~50 % of surveyed coastal households in 17 PICTs derived their first or second source of income from catching or selling fish.

However, there is limited research relating to two of the other goals, namely access to food and food utilisation. These goals are concerned with households’ ability to grow or buy foods to meet dietary requirements for good health [90]. The fourth goal, relating to stability of food systems, is beyond the scope of this literature review.

When planning policies and interventions to increase availability of fish, ease of access to fish, and utilization of fish in PICTs, it is important to consider other factors that may affect food intake. A lack of nutrition education and a decrease in knowledge of traditional food sources in several PICTs also means that many Pacific Islanders do not have the knowledge or skills to make food choices that will benefit their health [55, 59, 91, 92]. This is increasingly problematic as more imported unhealthy foods become readily available. Qualitative evidence suggests imported, foods such as canned meats may be increasingly preferred by Pacific Islanders over local foods, such as fresh fish, because they are associated with wealth and status [37]. The ability to purchase imported foods is increasing for at least two reasons. First, more PICT communities are moving away from a subsistence to a cash-based economy. Second, increased migration to urban centres reduces opportunities for people to grow their own food, or have access to fish when it is difficult to transport them from remote areas to urban markets.

One of the limitations of this review is that the reported findings of detailed studies on consumption of fish are based on only a few of the PICTs, and primarily in coastal areas. Evidence from many PICTs has been limited to information derived from household income and expenditure surveys [27], with little data on the factors determining access to fish and use of fish at a local level. In addition, the majority of articles included in this review were cross-sectional, with only one longitudinal study and one case–control study. Comparison of fish consumption was also limited because measurement of fish consumption differed between studies in terms of daily frequency, meals per month and daily amount. No intervention studies were found assessing the effect of fish consumption on improved dietary intakes or health outcomes.

Finally, a lack of co-ordinated approaches between fisheries and health ministries has been acknowledged by the Secretariat of Pacific Communities (SPC) [93]. This has hindered the development of integrated policies designed to achieve food security through improved management and sustainability of coastal fisheries, while at the same time preventing NCDs. Such approaches are a priority for the region and will require inter-sectoral dialogue and partnership.

## Conclusions

Recent literature confirms assumptions that fishing remains a major contribution to food security in Pacific Island countries and territories, through subsistence production and income generation (which allows food purchases). However, there is a paucity of research on how maintaining and/or improving fish consumption benefits the nutritional quality of the diets and health status of Pacific Islanders as they contend with the ongoing nutrition transition resulting from the influx of foods that are high in fat, sugar and salt. Given the significance of fish to food security and livelihoods, it is time for the region to develop a more integrated and coordinated approach to fisheries, health and food policy that identifies research priorities within national development challenges. A research and policy framework is required that addresses looming food security and public health crises caused by declining fisheries productivity, coastal fisheries management, increasing populations, and rising NCDs.

## Abbreviations

BMI: body mass index
NCDs: non communicable diseases
PICO: population, intervention, comparator, outcome
PICT: Pacific Island countries and territories
